# Isolated Fracture of the Posterior Tibial Margin: A Case Report

**DOI:** 10.7759/cureus.63662

**Published:** 2024-07-02

**Authors:** Achraf Tebbaa El Hassali, Mohammed Barrached, Adnane Lachkar, Najib Abdeljaouad, Hicham Yacoubi

**Affiliations:** 1 Orthopedics and Traumatology, Mohammed VI University Hospital, Faculty of Medicine and Pharmacy of Oujda, Mohamed I University, Oujda, MAR; 2 Orthopedics and Traumatology, Mohammed VI university hospital, Faculty of Medicine and Pharmacy of Oujda, Mohamed I University, Oujda, MAR

**Keywords:** posterior margin, syndesmosis, conservative treatment, distal, tibia, fracture

## Abstract

Isolated fractures of the distal end of the tibia are rare lesions; they can induce numerous complications and the diagnostic approach and management are not always simple. We report the case of a patient with an isolated fracture of the posterior margin of the tibia, exposing the different stages of its treatment compared to data from recent scientific literature.

## Introduction

The ankle is fundamental in maintaining static and dynamic body balance. It is a very stable joint whose main role is the transmission of movements and forces while walking. The posterior malleolus contributes significantly in the stability of the ankle and any disturbance can induce posterior subluxation of the talus [1].

Isolated fractures of the posterior marginal tibia are rare and initially described as “paratrooper fractures”, they represent only 0.5 to 1% of ankle fractures and there is a clear male predominance [2,3]. These fractures occur in an anatomical area where the tissue coverage is thin and particularly exposed, therefore with a high rate of complications [3].

## Case presentation

We report the case of a young patient aged 26, a student, non-athlete, and without any notable pathological history. He was the victim of a public road accident: He was a motorcyclist hit by a car with a point of impact at the right ankle level.

When he was admitted to the emergency room one hour after the accident, he was conscious, hemodynamically and respiratory stable. The osteoarticular examination found an edematous ankle, without skin opening or bruising. Palpation was painful and mobilization was possible but painful. The vascular and nervous examination were normal, pulses of posterior tibial and dorsalis pedis arteries were present with no cyanosis or coldness of the foot, and with a preserved foot sensibility. Standing position and active mobilization were made impossible because of the pain.

He benefited from an X-ray of the right ankle in anteroposterior and lateral views with an X-ray of the ipsilateral knee revealing a simple isolated and non-displaced fracture of the posterior marginal of the distal part of the tibia.

**Figure 1:**
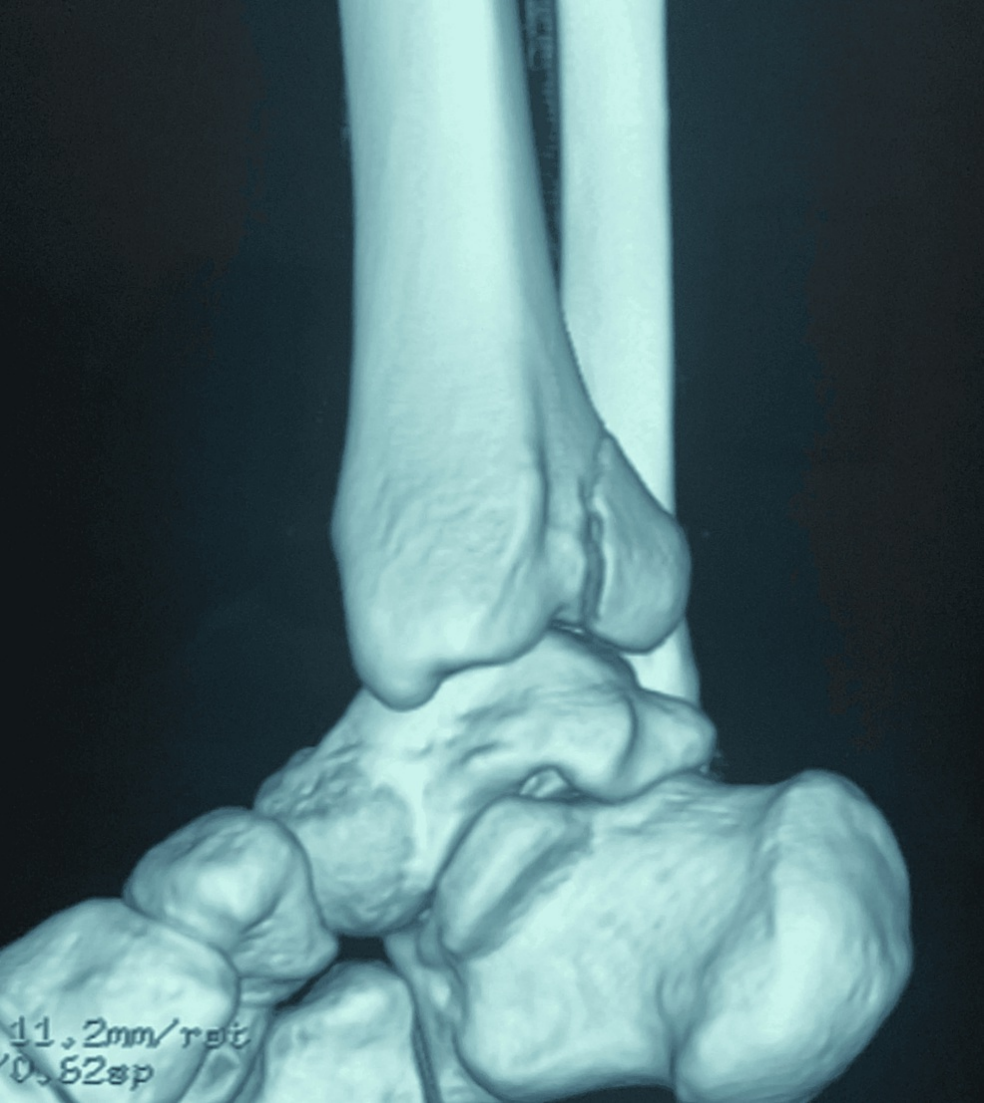
CT scan with a 3D reconstruction of the ankle showing the isolated fracture of posterior tibial margin

**Figure 2:**
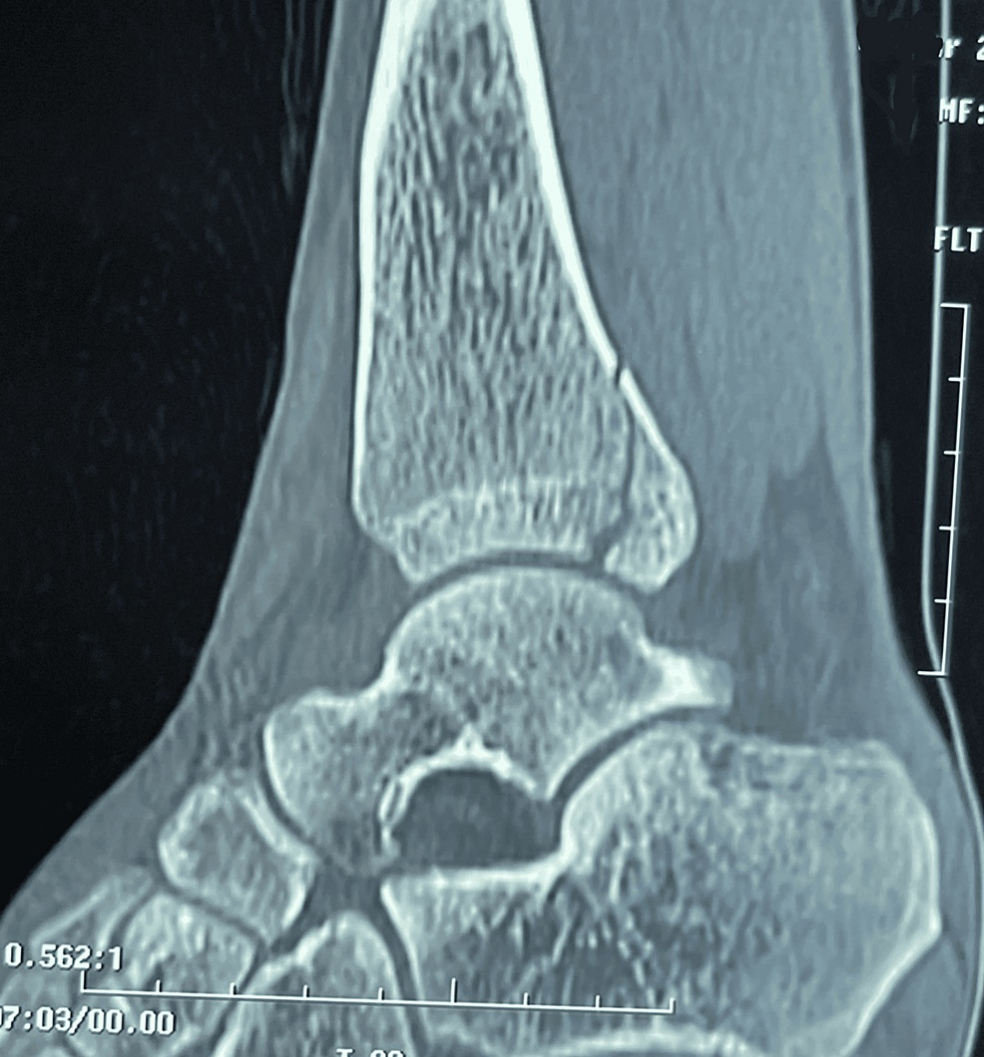
Sagittal section of the non-enhanced CT scan of the ankle confirming the isolated fracture of the posterior margin

**Figure 3:**
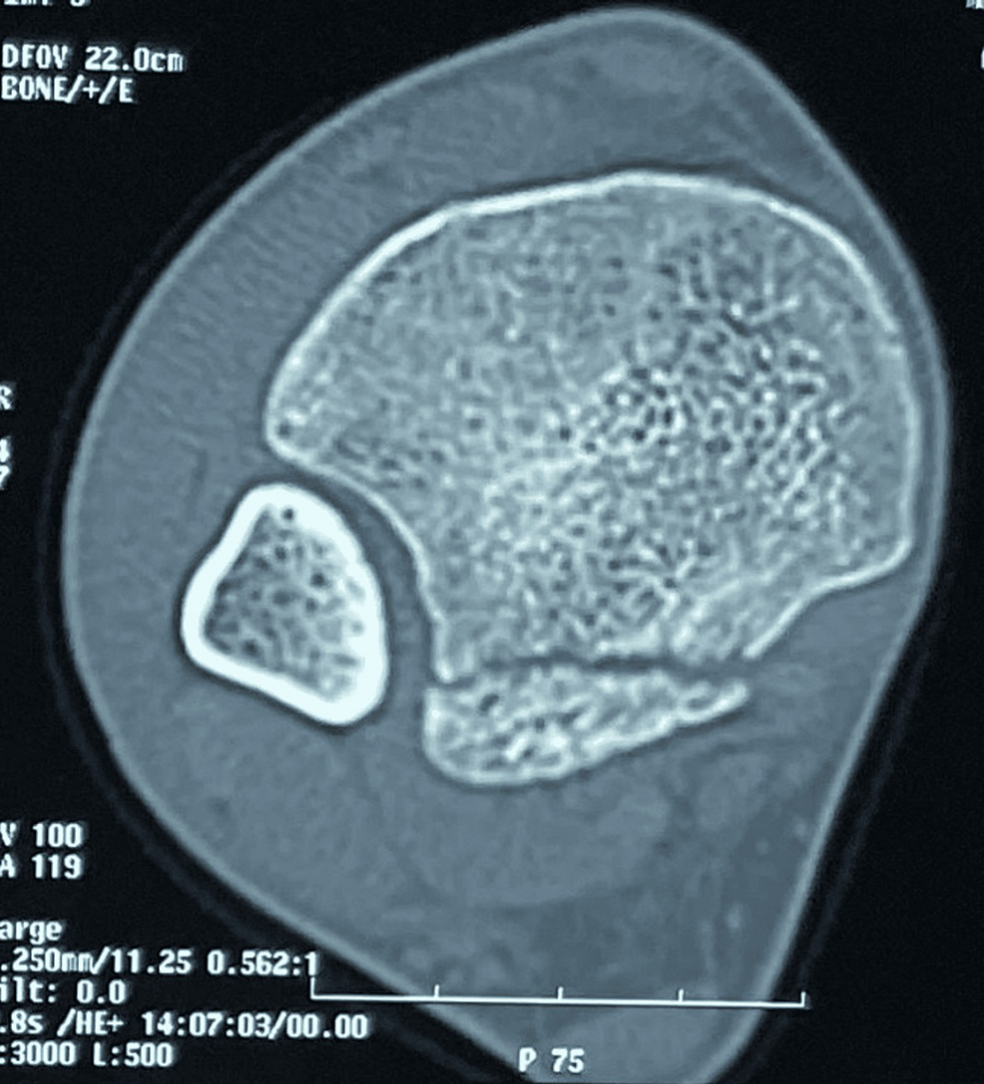
A transverse section of the non-enhanced CT scan of the ankle confirming the isolated fracture of the posterior margin

The radiological assessment was completed by a non-enhanced CT scan of the right ankle which confirmed isolated bone damage, without opening of the syndesmosis testifying to good joint congruence with less than 25% of the articular surface involved (Figures 1-3).

Faced with these elements, a non-surgical treatment was indicated for this patient consisting of immobilization with a cast boot, ankle at 90° for a period of 6 weeks with level II analgesic and anti-inflammatory treatment and thrombo-prophylaxis.

Removal of the cast was carried out after 6 weeks with gradual resumption of weight bearing and 20 sessions of physiotherapy made up of proprioception exercises, resumption of walking, physiotherapy, and recovery of joint range of motion. Follow-up of the patient for 6 months found a consolidated fracture without malunion with clinical symptoms: absence of pain, walking without lameness, and satisfactory range of motion.

## Discussion

Rare cases of isolated fractures of the posterior margin of the tibia have been reported, they are often associated with ligament damage. These are benign fractures mimicking an ankle sprain but cause serious complications and poor functional results [2,3].

Tomar et al. also reported the case of a young patient who, following a sprain while walking on uneven ground, who presented with an isolated fracture of the posterior marginal of the tibia associated with a probable rupture of the deltoid ligament. This fracture was treated surgically and the functional results were satisfactory [2]. Other cases of isolated fractures of the posterior marginal of the tibia with associated ligamentous damage have been reported in the literature by Smeeing et al. and Serbest et al. [3,4].

These fractures often result from axial loading on a fixed ankle in plantar flexion or rotational tension on the syndesmotic ligament [5]. The exact pathophysiological mechanism is not yet very clear [6]. Likewise, there is no specific classification.

Some rare classifications for isolated posterior marginal fractures based on CT scanning have been reported in the literature: Bartoníček et al. classified these fractures into five types as follows: Type 1) extraincisural fragment with an intact fibular notch; Type 2) posterolateral fragment extending into the fibular notch; Type 3) posteromedial two-part fragment involving the medial malleolus; Type 4) large posterolateral triangular fragment (involving more than one-third of the notch; Type 5) irregular osteoporotic fracture [7].

After careful clinical examination, standard X-rays of the ankle can be requested to individualize this type of fracture but the gold standard remains CT with 3D reconstructions which make it possible to highlight the fracture, study it, and visualize the lesions. associated with more appropriate management, and for certain ligament and tendon injuries, magnetic resonance imaging (MRI) may be necessary [8,9].

Despite the absence of specific recommendations for the treatment of isolated fractures of the posterior margin of the tibia, nevertheless several systematic reviews recommend conservative treatment in almost 87% of cases with satisfactory functional results. The therapeutic choice in this type of fracture is not always obvious but if it is surgical, it must then ensure bone reduction as precise as possible while respecting the surrounding tissues. In practice, it is often guided by the size of the posterior malleolar fragment, that is to say that the treatment must be conservative unless the fracture affects 25% or more of the articular surface or if there is a displacement of more than 2 mm, in these cases, surgery alone allows good reduction and fixation of the posterior fragment. The size of the posterior fragment and the therapeutic choice are not always correlated with the functional results obtained [8-11].

A recent study takes into consideration other factors for the choice of the therapeutic strategy such as the syndesmotic stability of the ankle, the residual subluxation of the talus, the joint congruence, and the involvement of the fibular notch [11] and several surgical fixation techniques such as fixation by anteroposterior screw, posteroanterior screw, and fixation by plate. The latter seems to be more effective according to a cadaveric study [12]. The functional results vary from one study to another and depend on the type of fracture, the associated lesions, and the management strategy.

## Conclusions

Isolated fractures of the posterior marginal tibia are rare and unique, they are prone to complications such as poor fracture repositioning, delayed fracture healing, and nonunion which makes them difficult to treat. They require detailed clinical and radiological analysis. Treatment, whether orthopedic or surgical, is sometimes challenging.
